# Comparison of Machine Learning Algorithms Identifying Children at Increased Risk of Out‐of‐Home Placement: Development and Practical Considerations

**DOI:** 10.1111/1475-6773.14601

**Published:** 2025-03-06

**Authors:** Tyler J. Gorham, Rose Y. Hardy, David Ciccone, Deena J. Chisolm

**Affiliations:** ^1^ IT Research & Innovation The Abigail Wexner Research Institute at Nationwide Children's Hospital Columbus Ohio USA; ^2^ Center for Child Health Equity and Outcomes Research The Abigail Wexner Research Institute at Nationwide Children's Hospital Columbus Ohio USA

**Keywords:** accountable care organization, machine learning, Medicaid, out‐of‐home placement, predictive modeling

## Abstract

**Objective:**

To develop a machine learning (ML) algorithm capable of identifying children at risk of out‐of‐home placement among a Medicaid‐insured population.

**Study Setting and Design:**

The study population includes children enrolled in a Medicaid accountable care organization between 2018 and 2022 in two nonurban Ohio counties served by the Centers for Medicare and Medicaid Services‐funded Integrated Care for Kids Model.

Using a retrospective cohort, we developed and compared a set of ML algorithms to identify children at risk of out‐of‐home placement within one year. ML algorithms tested include least absolute shrinkage and selection operator (LASSO)‐regularized logistic regression and eXtreme gradient‐boosted trees (XGBoost). We compared both modeling approaches with and without race as a candidate predictor. Performance metrics included the area under the receiver operating characteristic curve (AUROC) and the corrected partial AUROC at specificities ≥ 90% (pAUROC_90_). Algorithmic bias was tested by comparing pAUROC_90_ across each model between Black and White children.

**Data Sources and Analytic Sample:**

The modeling dataset was comprised of Medicaid claims and patient demographics data from Partners For Kids, a pediatric accountable care organization.

**Principal Findings:**

Overall, XGBoost models outperformed LASSO models. When race was included in the model, XGBoost had an AUROC of 0.78 (95% confidence interval [CI]: 0.77–0.79) while the LASSO model had an AUROC of 0.75 (95% CI: 0.74–0.77). When race was excluded from the model, XGBoost had an AUROC of 0.76 (95% CI: 0.74–0.77) while LASSO had an AUROC of 0.73 (95% CI: 0.72–0.74).

**Conclusions:**

The more complex XGBoost outperformed the simpler LASSO in predicting out‐of‐home placement and had less evidence of racial bias. This study highlights the complexities of developing predictive models in systems with known racial disparities and illustrates what can be accomplished when ML developers and policy leaders collaborate to maximize data to meet the needs of children and families.

**Summary**

- What is known on this topic
  ○ Out‐of‐home placements can promote child safety, but have been associated with adverse health conditions later in life.
  ○ Early intervention may help prevent unnecessary out‐of‐home placements, reducing adverse health outcomes and costs.
  ○ Machine learning algorithms can be developed to identify children at risk of out‐of‐home placement.
- What this study adds
  ○ The application of machine learning models to administrative data collected by governmental health and social services agencies can enhance the efficiency of risk prediction and account for unmeasured racial bias effects.

## 1 Introduction

The child welfare system, which includes foster care, endeavors to ensure the well‐being of children through the promotion of safe and supportive family environments [1]. The foster care system served over 600,000 children in the United States in calendar year 2021 at an estimated cost of over $2.8 billion [2]. Placement outside the home, while supporting child safety, is also associated with negative outcomes including poor mental health [3, 4, 5], lower educational attainment [4], chronic diseases like cardiovascular disease [3] and chronic obstructive pulmonary disease [6], and mortality [3]. Results have been mixed on whether placement outside the home or mandated support within the home is associated with greater adverse outcomes [7], however, there is general consensus that greater efforts should be made for placements to be the least restrictive possible [8]. The combined high cost and potential negative health effects of out‐of‐home placement make it a priority target for directed services that support early identification and intervention [1, 9].

Early identification requires an understanding of the precursors of out‐of‐home placement. While the reasons for removal from the home can be multifaceted and complex, neglect or abuse are among the most common [9]. Other reasons for placement outside the home include parental incarceration, family substance abuse, severe physical or behavioral health issues of the child, and voluntary relinquishment [1]. Studies attempting to model out‐of‐home placement have found significant associations with child factors including age, emotional and behavioral conditions, and developmental delays and adult factors including caregiver mental health, low caregiver social support, and previous reports of neglect or abuse [10, 11]. Ultimately, however, many of these models have low explanatory power as family situations are complex and decisions to place a child out of the home are multifactorial.

Out‐of‐home placement studies using data from the child welfare system have considered outcomes including placements among children with child protective services involvement [12], placement changes [13, 14], success of placements [15], and types of placements [14]. While valuable, these models focus on children already engaged in the child welfare system and thus do not inform primary prevention efforts. Fewer studies have assessed predictors of out‐of‐home placement in a broader pediatric population [10, 16, 17]. In one example, Brownell and colleagues [16] assessed predictors of out‐of‐home placements using a newborn screen for risk of out‐of‐home placement in Canada. They leveraged the use of multiple registries to assess the effectiveness of the screening in identifying children at risk for out‐of‐home placement. While valuable, this process required the collection of dedicated data with the associated logistical and cost challenges.

Medicaid insures approximately 39% of all children in the United States and, as such, is the largest insurer of children in the United States [18, 19]. Furthermore, they account for approximately $97.9 billion of Medicaid expenditures [18]. Given the evidence of negative outcomes associated with out‐of‐home placements, the cost associated with these placements, and the overarching goal of ensuring the health and wellbeing of all children, Medicaid has a special interest in identifying families at risk for out‐of‐home placement and finding ways to support these families to avoid such placements and ensure the health and well‐being of the children they serve.

By linking and employing administrative data from Medicaid and other social services systems along with geocoded community‐level indicators of social need, lower‐cost predictive modeling and machine learning have the potential to help proactively identify known and novel predictors and their relative importance. Such models have successfully predicted healthcare outcomes like hospital readmissions [20], risk of disease [21, 22], and care needs [23, 24]. They have also been used to predict social need outcomes such as experiences of homelessness [25]. Most validated models, to date, use data from adult populations due, in part, to considerations regarding the ethical use of artificial intelligence in pediatrics [26] and the challenges of employing multigenerational data. Opportunities exist for building such models in pediatric populations.

However, predictive modeling work needs to be considered within the context of the child welfare system and its well‐documented overrepresentation of Black, Native American, and Alaska Native children [27, 28]. In 2022, Black children accounted for 21% of children entering foster care while making up 14% of the US child population, and Native American or Alaska Native children accounted for 2% of the foster care population while representing 1% of the US child population [9, 29]. While there have been different theories as to the reason for this disproportionality, factors related to structural and systemic racism are frequently cited [28, 30, 31]. The Indian Child Welfare Act was passed in 1978 in response to the number of Native American children placed into non‐Native American homes, with the goal to further support Native families and their cultures [32]. For these historical reasons, understanding how machine learning algorithms may affect these historically marginalized populations, particularly with such a consequential outcome, is vital to ensuring that all children are in safe and supportive homes.

The promise of predictive modeling as a facilitator for proactive out‐of‐home placement intervention must be balanced with thoughtful consideration of concerns regarding interpretability [33], data confidentiality [34, 35, 36], and bias associated with modeling data collected within the context of child welfare systems that have disproportionately impacted Black, American Indian, and Native Alaskan families [28, 30, 37]. However, given the financial and social costs of out‐of‐home placement, high‐quality predictive models using existing secondary data to identify and serve youth at risk of out‐of‐home placement prior to engagement with the system could have substantial value. Our objective is to develop and test a set of interpretable machine learning algorithms capable of identifying children at risk of out‐of‐home placement among a Medicaid‐insured pediatric population.

## 2 Methods

### 2.1 Study Context

This study was conducted within the operations of the Ohio Integrated Care for Kids (InCK) program. The InCK model is an innovative service delivery and state payment model, funded through the Centers for Medicare and Medicaid Services, in seven demonstration programs in six states. Ohio's model focuses on Medicaid‐enrolled youth in two non‐urban Ohio Counties. InCK is designed to implement “a child‐centered local service delivery and state payment model that aims to reduce expenditures and improve the quality of care for children under 21 years of age covered by Medicaid through prevention, early identification, and treatment of behavioral and physical health needs.” [38] Ohio's current risk stratification model for early identification uses a rule‐based, expert‐derived, multi data source algorithm to assign Medicaid enrolled children to risk tiers. This paper describes an application of machine learning to refine this risk tiering process and enhance predictive accuracy.

### 2.2 Data and Sample

We performed a retrospective study analyzing Medicaid claims from Partners For Kids, an Ohio pediatric accountable care organization, focusing on individuals enrolled between 2018 and 2021 in the two Ohio InCK counties. While the InCK program targets those aged 0–20, our analysis was confined to participants under 18 to align with other research on out‐of‐home placements. Children were included in the analytic dataset without regard to history of out‐of‐home placements, and continuous Medicaid eligibility was not required to more accurately gauge the algorithm's effectiveness in the full Medicaid pediatric population.

### 2.3 Variables

A child's out‐of‐home placement was identified using Medicaid enrollment files. Children with either a plan class of “AFK” (adoption, foster, kindred care) or a child's Medicaid ID beginning with 089, which is assigned at the time of a custody episode, were coded as having an out‐of‐home placement starting in the month that the eligibility code changes. Importantly, Partners For Kids manages an individual‐level person identifier to limit loss‐to‐follow up related to changes in Medicaid ID that are often inherent to the foster care system. To approximate the model's application in identifying Medicaid‐eligible children as part of a monthly reporting procedure, we modeled a child's risk of changing to out‐of‐home placement status within one year of a theoretical monthly report based on Medicaid claims data, where data from the prior year are used to predict the risk of out‐of‐home placement over the next 12 months.

Our candidate predictors reflect criteria currently used in Ohio InCK's rules‐based, expert‐derived out‐of‐home placement risk assessment algorithm. This was done to allow for the possibility of a developed model to be deployed using existing program data, without the need for additional data collection. Due to challenges in linking parent and child Medicaid data, the model is limited to child‐level factors. Candidate predictors include history of out‐of‐home placement, past medical diagnoses categorized by the Pediatric Medical Complexity Algorithm [39], history of neonatal abstinence syndrome or substance use disorder diagnoses, a comprehensive behavioral health diagnoses indicator (Table S1), cumulative inpatient length of stay (e.g., total inpatient days in past year), and claims diagnosis codes serving as a proxy for eligibility for Ohio's Women, Infant, and Children (WIC) or Early Intervention programs (Tables S2 and S3). Additional community context indicators include Ohio Children's Opportunity Index (OCOI) quintiles and census tract‐level indicators of healthy food access and median household income provided by the USDA Food Access Research Atlas [40]. The OCOI is a census tract‐level composite measure of children's opportunity, based on 53 measures across the following domains: family stability, infant health, children's health, access to local services, education, housing stability and affordability, natural and built environment, and criminal justice (e.g., likelihood of victimization) [41]. Higher quintiles represent higher levels of opportunity.

### 2.4 Statistical Analyses

#### 2.4.1 Machine Learning Algorithm Development

We developed four separate machine learning algorithms designed to predict a child's one‐year risk of out‐of‐home placement. These models included least absolute shrinkage and selection operator (LASSO)‐regularized logistic regression and eXtreme Gradient Boosting (XGBoost) decision tree models, with and without the inclusion of child's race as a candidate predictor. The inclusion of race was varied to understand how considering race influenced model bias and predictive performance. LASSO was selected as one of our two approaches for its strengths in interpretability (complete transparency in how the final model generates its predictions) and variable selection (a model's ability to identify useful candidate predictors and eliminate those that do not significantly improve model performance). XGBoost is a boosted decision tree machine learning framework that, when coupled with model‐agnostic explainers such as Shapley additive explanation (SHAP) values, provides a balance of interpretability and potentially greater predictive performance than what can be achieved by more traditional statistical approaches.

LASSO models were trained using glmnet [42] in R version 4.2.2 [43], while XGBoost models were trained using python's scikit‐learn [44] and XGBoost [45] packages. SHAP values were calculated using the tree explainer as part of the SHAP [46] package and were plotted using Matplotlib [47]. Receiver operating characteristic curves were built using ggplot2 [48] in R.

### 2.5 Performance Metrics

Area under the receiver operating characteristic curve (AUROC) and standardized, partial AUROC at specificities ≥ 90% (pAUROC_90_) were calculated to compare models. The AUROC is a common metric that reflects a model's balance of sensitivity and specificity. The pAUROC_90_ represents a model's performance among those children identified as being at increased risk of out‐of‐home placement at a decision threshold that allows for a conservative specificity of 90% (equal to a false positive rate of 10%). Our current expert‐derived risk stratification algorithm places roughly 12% of children at higher risk for out‐of‐home placement and thus eligible for more prevention interventions, such as care coordination services. A 10% of these children have not been placed out of the home in the past 18 months, so we consider a false positive rate of 10% acceptable in our predictive model explorations.

Model performance for both LASSO and XGBoost models was estimated using 10‐fold cross‐validation during model development. AUROC and pAUROC_90_ confidence intervals (CI), as well as pairwise comparisons between ROC curves, were constructed via 10,000 bootstrap samples from the cross‐validated predictions using the pROC [49] package in R.

### 2.6 Racial Bias Investigation

A subgroup analysis was conducted, comparing each model's pAUROC_90_ when scoring Black and White patients separately. We observed fewer than 20 total out‐of‐home placements among children whose race was identified as Asian, Native American, and other/unknown, therefore our analysis of racial bias performance was limited to Black or African American and White children. This subgroup approach was inspired by Obermeyer et al. [50] but relies on comparing pAUROC_90_ as a means of evaluating model performance for all potential decision thresholds at or above 90% specificity. This approach is similar to the concept of equal opportunity (which compares a model's sensitivity or true positive rate at a given specificity) without requiring a single, potentially arbitrary operating threshold, as this would be decided by stakeholders during model deployment.

This study was approved by the Nationwide Children's Hospital Institutional Review Board (STUDY00001707) as research presenting no greater than minimal risk.

## 3 Results

Our final modeling population included 31,518 children, representing 833,557 unique member‐months (Table 1). Our median age was 8 years old (range: 0–17) and 48% were classified as female. This population was 69% White, 9% Black or African American, 4% Asian, and 0.3% Native American, with 18% of children with unknown or missing race.

**TABLE 1:** Study population by out‐of‐home placement status.

	Never placed out‐of‐home	At least one out‐of‐home placement
Individuals (*N*, %)	31,191 (99.0)	327 (1.0)
Person‐months (*N*, %)	828,087 (99.3)	5470 (0.7)
Age in years (mean, SD)^a^	8.0 (5.1)	6.8 (4.9)
Gender (*N*, %)		
Female	15,079 (98.9)	163 (1.1)
Male	16,076 (99.0)	164 (1.0)
Unknown	36 (100)	0 (0)
Race (*N*, %)		
African American	2678 (98.4)	43 (1.6)
Asian	1191 (99.9)	—
Native American	80 (97.6)	—
Other or unknown	5629 (99.7)	15 (0.3)
White	21,613 (98.8)	266 (1.2)

^a^ Age statistics are represented at the person‐month level where a child's age will be represented once for every month of Medicaid eligibility. Cells with *N* < 10 have been suppressed to preserve privacy.

### 3.1 Predictive Performance

**FIGURE 1:**
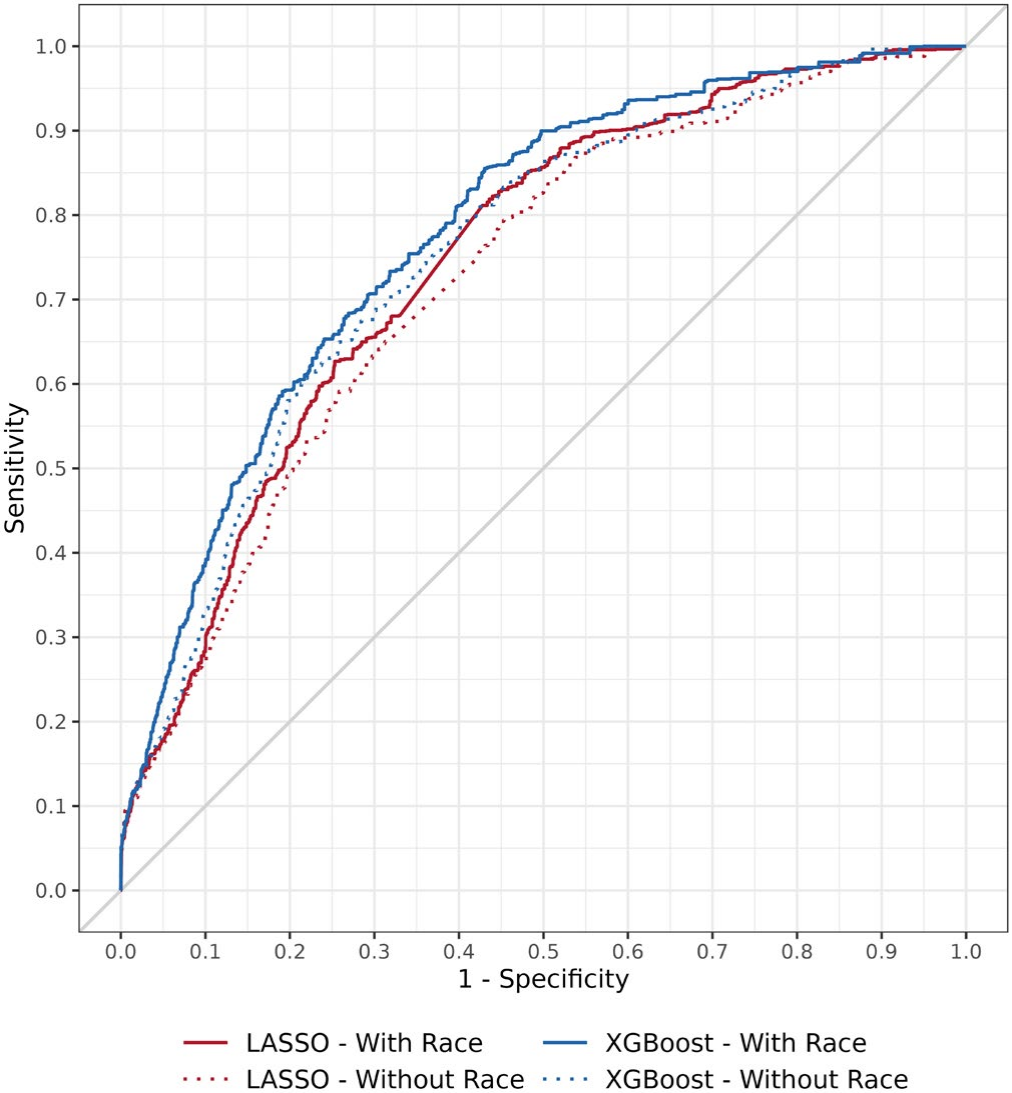
Receiver operating characteristic curves. Overall, XGBoost models outperformed LASSO models, and within algorithm type, models including race performed better than those without race.

Overall, XGBoost models outperformed LASSO models, both with and without race as a candidate model criterion (Figure 1). XGBoost with race was the best performing model in terms of both AUROC (0.78, 95% CI: 0.77–0.79) and pAUROC_90_ (0.60, 95% CI: 0.59–0.60). The second‐best performing model was XGBoost without race (0.76, 95% CI: 0.74–0.77; *p* < 0.001 when compared to XGBoost with race), followed by LASSO with race (0.75, 95% CI: 0.74–0.77), and LASSO without race (0.73, 95% CI: 0.72–0.74; *p* < 0.001 when compared to LASSO with race). XGBoost with race significantly outperformed both the LASSO model with race (pAUROC_90_ 0.57, 95% CI: 0.56–0.58; *p* < 0.001) and the XGBoost model without race (pAUROC_90_ 0.58, 95% CI: 0.57–0.59; *p* < 0.001). No significant differences in pAUROC_90_ were identified in pairwise comparisons among the three remaining models.

### 3.2 Feature Importance

There was good agreement between predictors identified in the two LASSO models (Table S4). The highest coefficient values for both LASSO models included history of neonatal abstinence syndrome, history of out‐of‐home placement, and history of substance use disorder. Additionally, living in a census tract within the highest two quintiles for OCOI's Access domain was the strongest protective factor across both LASSO models, with the exception of Asian race in the model that included race.

For both XGBoost models, younger age and history of past out‐of‐home placement were consistently associated with a higher predicted risk of out‐of‐home placement (Figures S1 and S2). Within the XGBoost model with race, Black or African American children generally had a higher predicted risk of out‐of‐home placement, while Asian children had consistently lower predicted risk scores (Figure S2).

### 3.3 Bias Evaluation

**FIGURE 2:**
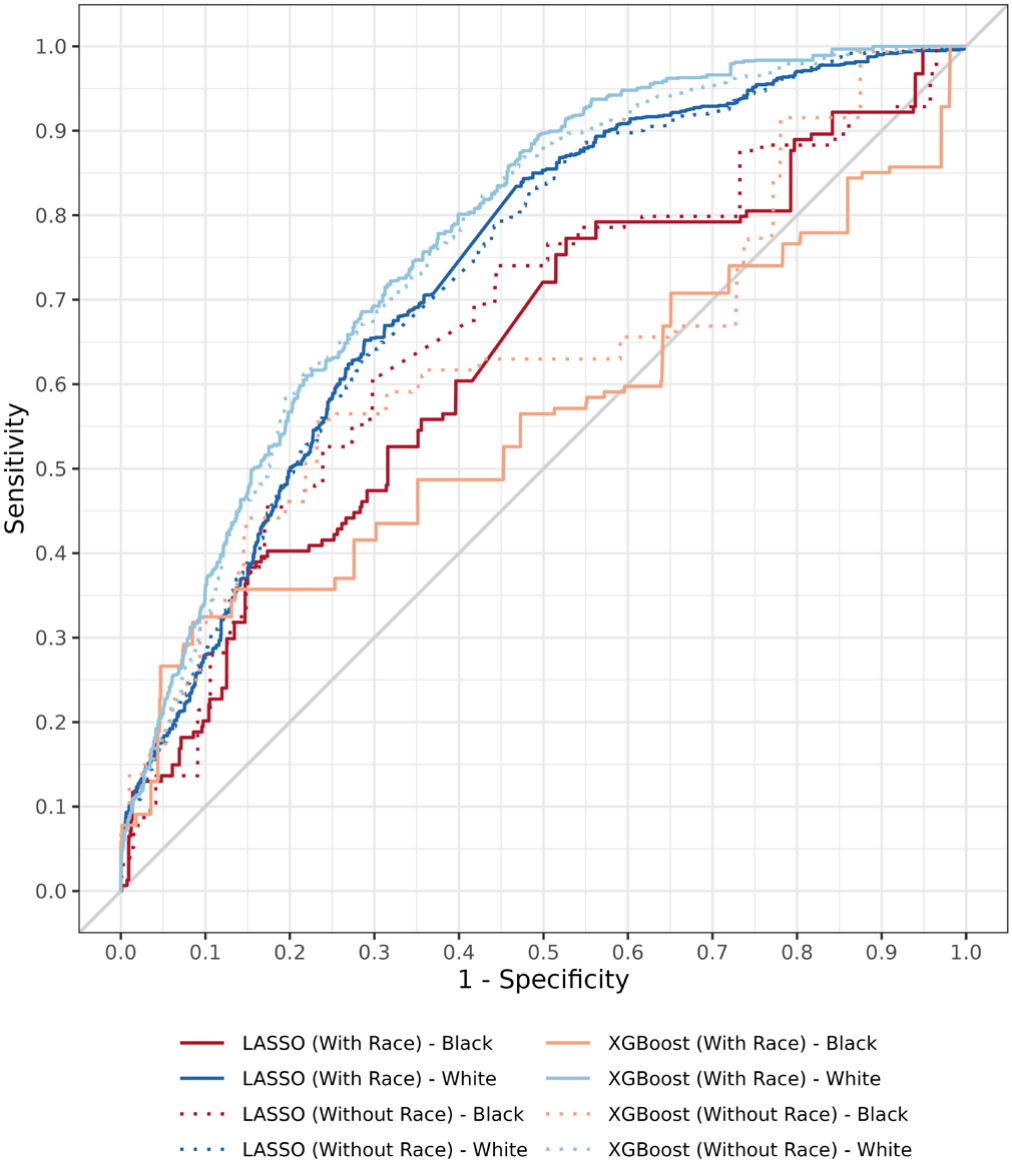
Receiver operating characteristic curves by race. While model performance is similar at very high specificities (where the models would be used in practice), there is a concerning trend of better model performance among White children than Black children for every algorithm tested when considering the full range of model predictions.

Testing the four models on Black and White subpopulations yielded potentially biased results for our simplest model, LASSO without race, where the pAUROC_90_ for Black children was 0.536 (0.514–0.562) compared to 0.567 (0.556–0.576) among White children (*p* = 0.02; Figure 2). No significant differences in pAUROC_90_ were identified between Black and White children in the remaining models (Table 2). Bias evaluations were limited to Black and White children due to the very small number of out‐of‐home placements among other racial groups (Table 1).

**TABLE 2:** Model performance among Black children and White children.

	Black—pAUROC_90_ (95% CI)	White—pAUROC_90_ (95% CI)	*p*
LASSO, without race	0.536 (0.514–0.562)	0.567 (0.556–0.576)	0.02
LASSO, with race	0.545 (0.522–0.571)	0.568 (0.558–0.578)	0.11
XGBoost, without race	0.575 (0.547–0.606)	0.574 (0.565–0.583)	0.95
XGBoost, with race	0.580 (0.552–0.606)	0.583 (0.573–0.593)	0.81

## 4 Discussion

Identification of children at risk of out‐of‐home placement is challenging but critical to primary prevention work and intervention efforts to prevent adverse outcomes associated with out‐of‐home placements. Our study aimed to develop an interpretable and translatable machine learning algorithm using Medicaid claims data that could be used by others interested in such primary prevention work. In support of that goal, we trained four machine learning algorithms aimed at identifying children at risk of out‐of‐home placement within one year. With just child Medicaid claims and community‐level social determinants of health characteristics, our models' predictive performances were comparable to others being used in the field that use behavioral assessment and child demographic data [15]. This suggests that even with limited information, our models' predictive performance is within the range of other, more resource intensive tools being used.

Although there were some differences between the LASSO and XGBoost models, many predictive factors were consistent with those found in the literature. For example, both the LASSO and XGBoost models found that a history of out‐of‐home placement and younger age were predictive of future placements. This finding aligns with that of English and colleagues [10] who found that past reports of abuse or neglect were associated with out‐of‐home placements.

While our models did not include family and household characteristics, some of the variables found to be important in our models suggest that such characteristics are important and align with previous literature. Similar to other models that have found that caregiver factors like mental health conditions, substance use, financial difficulties, and a parent's own history of abuse or neglect were important predictive factors [10, 13, 16], our LASSO models found that a history of neonatal abstinence syndrome, representing in utero exposure by way of a substance using birth parent, was predictive. Notably, our findings indicate that substance use, either by the caregiver or by the child, is an important risk factor. While family and household characteristics are not included, we do find that children residing in neighborhoods with greater geographic access to local services like healthcare providers, supermarkets, and schools were less likely to be at risk of out‐of‐home placements. This finding provides further evidence of the importance of such local services and provides potential targets for future research and primary prevention interventions.

Literature has shown racial disproportionalities within child welfare systems as well as racial disparities in related outcomes. This motivated our racial bias analyses [27, 28]. Not only are Black children more likely to be represented in the child welfare system, but there is evidence that Black children are more likely to be placed outside the home [51] and remain in foster care for longer [52, 53]. There is mixed evidence that they are less likely to be reunified with families [54, 55, 56] which may be due to differences in state policies [56]. Some have suggested that the racial disproportionality and outcome disparities are a result of lower socio‐economic status. However, among children receiving public assistance, Black children were overrepresented in child welfare and underrepresented in being referred for family support services, suggesting that lower socio‐economic status is not the only reason for this disproportionality.

In our investigation of the racial biases of these models, we find model thresholds leading to high specificities, greater than 90%, where the model would be most likely to be deployed, three of our four models did not display statistically significant evidence of racial bias when taking all potential thresholds together (the so‐called partial area under the curve). We emphasize this region of the receiver operating characteristic curve because specificity thresholds below 90% would lead to too many false positives and could be considered an inefficient use of intervention‐related resources. The best performing model was trained using XGBoost and included children's race as a candidate predictor, with nearly identical model performance between Black and White children at specificities greater than 90%. This inclusion of race as an approach to mitigating algorithmic bias (i.e., a racially conscious modeling approach) provides further evidence against the idea of “fairness through unawareness.” The inclusion of race also reduced the significant racial bias originally found in our LASSO model between Black children and White children (Table 2). While we did not observe significant evidence of racial bias when considering the overall partial areas under the curve for three models, it would be critical for specific model thresholds to be evaluated before being used as decision thresholds in a deployed algorithm, as equality of opportunity [57] may still be violated for a specific threshold, even when partial areas under the curve are similar. For example, we observe visual differences in model sensitivity for both of our LASSO models (but neither XGBoost model) at a specificity of 90% (Figure 2).

Overall, our findings support the inclusion of race as a candidate variable when known or observed risk factors related to race may contribute to inequities in the outcome being predicted, particularly when additional data that could account for observed racial disparities is unavailable. After potential models have been tested with and without race as a candidate variable, model developers should work with stakeholders and the individuals impacted by model predictions to consider the potential implications of the inclusion of race within the model and whether observed model performance improvements, if any, outweigh potential negative implications of including race within the final model. A similar case could be made for the use of a more complex and less explainable modeling approach, such as XGBoost, compared to fully interpretable models, where a tradeoff between interpretability of simpler models and potential gains in model performance by more complex models–even reduced racial bias, as demonstrated here–should be discussed with individuals outside the model building team.

In the case of the models under study here, we found the inclusion of race as a candidate predictor within the LASSO modeling approach lessened the gap in predictive model performance when applied to Black and White children, potentially by capturing unmeasured factors that contribute to a child's risk of being placed out of the home that differ between Black and White children. Notably, the more complex XGBoost approach had a narrower racial disparity at high specificities than LASSO (no significant difference observed), but this only holds true at specificities greater than 85%. Whether this is due to our unbalanced training data (75% White vs. just 9% Black), which may allow the algorithms to learn more about patterns of out‐of‐home placement risk factors that impact White children or some other cause is unknown.

When observing the complete ROC curves, we see that regardless of whether race is included in the model, our predictive modeling appears to predict out‐of‐home placement more accurately among White children enrolled in Medicaid compared to Black children enrolled in Medicaid. This may suggest that the child and community level variables available to our model are more strongly related to out‐of‐home placement for White children and that data on unmeasured parental, family, and system factors (e.g., unconscious bias, disproportional parental incarceration) would be required for a stronger model in Black children. Understanding the reasons behind racial differences in out‐of‐home placements is critical as we seek to reduce out‐of‐home placements across the country.

### 4.1 Limitations

Our study includes a few important limitations. First, this analysis was situated within our ongoing work with the InCK model. As such, we focus on just two primarily rural counties in central Ohio (US Midwest). There is limited racial and ethnic diversity within these counties, and specific factors contributing to out‐of‐home placement, as identified in our models, may not translate outside of this region. Predictors and variable weights would need to be adjusted for other populations. Second, this work was limited to children enrolled in Medicaid, who are predominately eligible due to their family's low‐income. As such, its generalizability to all children is limited. However, as Medicaid covers approximately 39% of US children [18, 19], it still provides important information on a substantial group of children. Third, additional work is needed to characterize potential racial biases before serious considerations of model implementation could begin. While our approach of relying on pAUROC_90_ did not identify statistically significant differences in model performance between Black and White children at high specificities, a simple visual inspection of Figure 2 suggests additional bias mitigation techniques may be warranted. This may include identifying data sources that better capture risk factors of out‐of‐home placement for Black children or oversampling Black children and other non‐White racial groups during model training. This is the next step in our work.

Finally, these models used factors related to the child and community‐level social determinants of health that could be measured from Medicaid claims data and area‐level indices of opportunity. The next step for this work is to further refine and validate the model with the inclusion of social service data currently not available under our project‐specific state agency data use agreement. Ultimately, we intend to test whether referring children with identified moderate to high risk to care support services can reduce the probability of out‐of‐home placement.

### 4.2 Policy, Practice, and Research Implications

Meeting the needs of children at risk of out‐of‐home placement requires understanding those needs and facilitating linkages to the systems that can address them. This is the purpose of the InCK model. Identifying those at risk is challenging, however, because the optimal data needed is controlled by multiple state and local agencies with limited mechanisms for data sharing. This analysis shows that models with good predictive value can be achieved with just Medicaid administrative data enhanced by publicly available community‐level data. These models can then be used to proactively offer family‐centered case management at the individual level. Furthermore, geographic information on at‐risk youth identified through these models can highlight areas where community‐ and school‐based interventions may be most beneficial. Using machine learning models, designed with a conscious recognition of the need for bias mitigation, can get the right resources to the right families at the right time, which is the key to prevention and health equity.

As we consider the ethical use of predictive modeling, this work highlights the complexities of using race in our models. It requires thoughtful consideration of why race might be included, the mechanisms by which race may be a proxy for larger systemic biases, and the ways the results could be interpreted. Numerous organizations are standing against race‐based medicine and altering long‐established clinical score calculations that include race due to racist ideas [58, 59]. This is to be celebrated. However, when known health disparities exist for an outcome as a result of structural racism, the inclusion of race in predictive models may accurately capture the indirect effects of that racism on the studied outcome. This race‐conscious [60] approach requires deliberate consideration of the relationships between race and the target outcome and whether the inclusion of race in a predictive model may promote health equity or exacerbate existing racial disparities [61].

There are opportunities for predictive modeling to provide less time‐ and resource‐intensive methods to identify families in need of support. There are, however, many practical considerations before the deployment of such a model could be implemented. As we think about predictive modeling in the real world, balancing the model performance, interpretability, and potential racial biases are just a few considerations. If ML‐based model performance is similar to expert‐driven algorithms, we need to consider what is gained from ML models and to what degree each approach adds value to the goals of the project. Additionally, data sharing across multiple partners and with multiple data sources can be incredibly challenging, and so balancing those challenges with the value that these data sources add to model performance is important. Finally, we find that consideration of racial biases, available resources, and experiences of those affected by these models are critical to determining the thresholds at which such models would be deployed in the real world.

## 5 Conclusions

This work demonstrates that developing a less time‐ and resource‐intensive tool using predictive modeling to identify out‐of‐home placement is possible while highlighting the complexities of development and implementation. We observed that the slightly more complex approach of XGBoost outperformed the more interpretable LASSO, and that the inclusion of race as a candidate predictor significantly improved results for both approaches. We further find that local services may be an important and modifiable factor associated with out‐of‐home placements, a consideration for future interventions. Navigating critical interpretability and ethical considerations before model implementation in the real world is possible with the help of machine learning algorithm developers and policy leaders.

## Conflicts of Interest

The authors declare no conflicts of interest.

## Supporting information


Data S1.

## Data Availability

To preserve patient privacy, research data are not shared.
