# Supplementary material for: Comparison of Machine Learning Algorithms Identifying Children at Increased Risk of Out‐of‐Home Placement: Development and Practical Considerations
Source: Health Serv Res. 2025 Mar 6;60(4):e14601. doi: 10.1111/1475-6773.14601 (PMC12277119; doi:10.1111/1475-6773.14601)
Supplement: Supplementary file 1 — Data S1. [file HESR-60-0-s001.docx]

**Supplemental Table 1.** Comprehensive Behavioral Health Definition

| Criteria | | Set | Additional Restrictions |
| --- | --- | --- | --- |
| Place of Service | | **POS:**  '55' | **CDE_CLM_TYPE:** 'A', 'B', 'C', 'I', 'M', 'O' |
| Billing Provider Type | | **DTL_CDE_PROV_TYPE_PRI_BILL:**  '42', '84', '95' | **CDE_CLM_TYPE:** 'B', 'M' |
| DRG | | **DRG:**  '740', '750', '751', '752', '753', '754', '755', '756', '757', '758', '759', '760', '770', '773', '774', '775', '776' | **CDE_CLM_TYPE:** 'A', 'I' |
| Revenue Code | | **RevCode:**  '0114', '0116', '0124', '0126', '0134', '0136', '0144', '0146', '0154', '0156', '0204', '0513', '0671', '0900', '0901', '0902', '0903', '0904', '0905', '0906', '0907', '0909', '0910', '0911', '0912', '0913', '0914', '0915', '0916', '0917', '0918', '0919', '0944', '0945', '0961', '1000', '1001', '1002', '1003', '1004', '1005', '1006' | **CDE_CLM_TYPE:** 'C', 'O' |
| ICD-10 Primary Diagnosis Code | | **substr(ICDDiag1,1,2):**  'F0', 'F2', 'F3', 'F4', 'F5', 'F6', 'F9', 'X7', 'X8' **substr(ICDDiag1,1,3):** 'F10', 'F11', 'F12', 'F13', 'F14', 'F15', 'F16', 'F18', 'F19', 'F84', 'F88' **substr(ICDDiag1,1,4):** 'Z915' **substr(ICDDiag1,1,5):** 'R4585', 'Z7281', 'T1491' **substr(ICDDiag1,1,6):** 'T71112', 'T71122', 'T71132', 'T71152', 'T71162', 'T71192', 'T71222', 'T71232' | **CDE_CLM_TYPE:** 'A', 'B', 'C', 'I', 'M', 'O' |
| Healthcare Common Procedure Coding System (HCPCS) | | **Behavioral Health Services HCPCS:**  '90785', '90791', '90792', '90801', '90804', '90806', '90808', '90810', '90812', '90814', '90832', '90833', '90834', '90836', '90837', '90838', '90839', '90840', '90846', '90847', '90849', '90853', '90863', '90899', '96101', '96105', '96110', '96111', '96115', '96116', '96117', '96118', '96127', '99406', '99407', 'G0396', 'G0397', 'H0001', 'H0003', 'H0004', 'H0005', 'H0006', 'H0007', 'H0010', 'H0011', 'H0012', 'H0014', 'H0015', 'H0036', 'H0038', 'H0040', 'H0046', 'H0047', 'H0048', 'H2012', 'H2015', 'H2017', 'H2019', 'H2020', 'H2034', 'H2036', 'S0201', 'S0281', 'S9484'  **Office Administered Drugs HCPCS:**  'J0400', 'J0401', 'J0570', 'J0571', 'J0571', 'J0573', 'J0574', 'J0575', 'J1630', 'J1631', 'J2315', 'J2358', 'J2426', 'J2680', 'J2794', 'J3360', 'H0020' | **CDE_CLM_TYPE:** 'A', 'B', 'C', 'I', 'M', 'O'  Non-ED |
| HCPCS + National Drug Code (NDC) | | **HCPCS = 'J8499' and NDC is one of the following:** '63459030042', '65757030202', '00406009201', '00406009203', '00185003901', '00185003930', '00406117001', '00406117003', '00555090201', '00555090202', '16729008101', '16729008110', '42291063230', '43063059115', '47335032683', '47335032688', '50436010501', '51224020630', '51224020650', '52152010502', '52152010504', '52152010530', '54868557400', '65694010003', '65694010010', '68084029111', '68084029121', '68094085362', '68115068030', '00056001122', '00056001130', '00056001170', '00056007950', '51285027501', '51285027502', '00056008050', '65757030001', '00054017613', '00054017713', '00093537856', '00093537956', '00228315303', '00228315603', '00378092393', '00378092493', '50383092493', '50383093093', '35356055530', '35356055630', '42858050103', '42858050203', '43063075306', '55700030230', '55700030330', '62756045983', '62756046083', '68308020230', '68308020830', '12496131002', '12496010001', '12496010002', '12496010005', '12496030001', '12496030002', '12496030005' | **CDE_CLM_TYPE:** 'A', 'B', 'C', 'I', 'M', 'O'  Non-ED |
| Therapeutic Class | | **CDE_THERA_CLS_SPEC:**  'C0D', 'G6A', 'H2F', 'H2G', 'H2H', 'H2L', 'H20', 'H2O', ''H2S', 'H2U', 'H2V', 'H3T', 'H3W', 'H4B', 'H7B', 'H7C', 'H7D', 'H7E', 'H7J', 'H7O', 'H7P', 'H7R', 'H7S', 'H7T', 'H7U', 'H7V', 'H7W', 'H7X', 'H7Y', 'H8M', 'H8P', 'H8R', 'H8S', 'H8T', 'H8W', 'H8Y', 'J5B' | **CDE_CLM_TYPE:** 'P', 'Q' |
| Place of Service (POS) | **POS:**  '55' | | **CDE_CLM_TYPE:** 'A', 'B', 'C', 'I', 'M', 'O' |
| Billing Provider Type | **DTL_CDE_PROV_TYPE_PRI_BILL:**  '42', '84', '95'c | | **CDE_CLM_TYPE:** 'B', 'M' |
| DRG | **DRG:**  '740', '750', '751', '752', '753', '754', '755', '756', '757', '758', '759', '760', '770', '773', '774', '775', '776' | | **CDE_CLM_TYPE:** 'A', 'I' |
| Revenue Code | **RevCode:**  '0114', '0116', '0124', '0126', '0134', '0136', '0144', '0146', '0154', '0156', '0204', '0513', '0671', '0900', '0901', '0902', '0903', '0904', '0905', '0906', '0907', '0909', '0910', '0911', '0912', '0913', '0914', '0915', '0916', '0917', '0918', '0919', '0944', '0945', '0961', '1000', '1001', '1002', '1003', '1004', '1005', '1006' | | **CDE_CLM_TYPE:** 'C', 'O' |
| ICD-10 Primary Diagnosis Code | **substr(ICDDiag1,1,2):**  'F0', 'F2', 'F3', 'F4', 'F5', 'F6', 'F9', 'X7', 'X8' **substr(ICDDiag1,1,3):** 'F10', 'F11', 'F12', 'F13', 'F14', 'F15', 'F16', 'F18', 'F19', 'F84', 'F88' **substr(ICDDiag1,1,4):** 'Z915' **substr(ICDDiag1,1,5):** 'R4585', 'Z7281', 'T1491' **substr(ICDDiag1,1,6):** 'T71112', 'T71122', 'T71132', 'T71152', 'T71162', 'T71192', 'T71222', 'T71232' | | **CDE_CLM_TYPE:** 'A', 'B', 'C', 'I', 'M', 'O' |
| Procedure Code | **BH Services HCPCS:**  '90785', '90791', '90792', '90801', '90804', '90806', '90808', '90810', '90812', '90814', '90832', '90833', '90834', '90836', '90837', '90838', '90839', '90840', '90846', '90847', '90849', '90853', '90863', '90899', '96101', '96105', '96110', '96111', '96115', '96116', '96117', '96118', '96127', '99406', '99407', 'G0396', 'G0397', 'H0001', 'H0003', 'H0004', 'H0005', 'H0006', 'H0007', 'H0010', 'H0011', 'H0012', 'H0014', 'H0015', 'H0036', 'H0038', 'H0040', 'H0046', 'H0047', 'H0048', 'H2012', 'H2015', 'H2017', 'H2019', 'H2020', 'H2034', 'H2036', 'S0201', 'S0281', 'S9484' **Office Administered Drugs HCPCS:**  'J0400', 'J0401', 'J0570', 'J0571', 'J0571', 'J0573', 'J0574', 'J0575', 'J1630', 'J1631', 'J2315', 'J2358', 'J2426', 'J2680', 'J2794', 'J3360', 'H0020' | | **CDE_CLM_TYPE:** 'A', 'B', 'C', 'I', 'M', 'O'  Non-ED |
| HCPCS + NDC | **HCPCS = 'J8499' and NDC is one of the following:** '63459030042', '65757030202', '00406009201', '00406009203', '00185003901', '00185003930', '00406117001', '00406117003', '00555090201', '00555090202', '16729008101', '16729008110', '42291063230', '43063059115', '47335032683', '47335032688', '50436010501', '51224020630', '51224020650', '52152010502', '52152010504', '52152010530', '54868557400', '65694010003', '65694010010', '68084029111', '68084029121', '68094085362', '68115068030', '00056001122', '00056001130', '00056001170', '00056007950', '51285027501', '51285027502', '00056008050', '65757030001', '00054017613', '00054017713', '00093537856', '00093537956', '00228315303', '00228315603', '00378092393', '00378092493', '50383092493', '50383093093', '35356055530', '35356055630', '42858050103', '42858050203', '43063075306', '55700030230', '55700030330', '62756045983', '62756046083', '68308020230', '68308020830', '12496131002', '12496010001', '12496010002', '12496010005', '12496030001', '12496030002', '12496030005' | | **CDE_CLM_TYPE:** 'A', 'B', 'C', 'I', 'M', 'O'  Non-ED |
| Therapeutic Class | **CDE_THERA_CLS_SPEC:**  'C0D', 'G6A', 'H2F', 'H2G', 'H2H', 'H2L', 'H20', 'H2O', ''H2S', 'H2U', 'H2V', 'H3T', 'H3W', 'H4B', 'H7B', 'H7C', 'H7D', 'H7E', 'H7J', 'H7O', 'H7P', 'H7R', 'H7S', 'H7T', 'H7U', 'H7V', 'H7W', 'H7X', 'H7Y', 'H8M', 'H8P', 'H8R', 'H8S', 'H8T', 'H8W', 'H8Y', 'J5B' | | **CDE_CLM_TYPE:** 'P', 'Q' |
| Note: ED is defined as (CDE_CLM_TYPE in('C','O') and substr(RevCode,1,3) = '045') or (CDE_CLM_TYPE in('B','M') and HCPCS in('99281', '99282', '99283', '99284', '99285', '99288')). | | | |

**Supplemental Table 2. Women, Infant, and Children** **Program Eligibility Proxy**

| Eligibility Criteria | ICD-9, ICD-10 Claim Code |
| --- | --- |
| Born early (<39 weeks) and less than 2 years old | Z3A.08-Z3A-38 |
| Failure to Thrive | R62.51, P92.6 |
| Lactose Intolerance | E73.9 |
| Developmental, Sensory, Motor Disability | F80*, F81*, F82, F84*, F88, F89, R62*, F32.0,  F32.1,  F32.2,  F32.3, F32.4, F32.5, F32.8, F32.9, F33.0, F33.1, F33.2, F33.3, F33.40, F33.41,F33.42, F33.8, F33.9, F34.1, F43.21, F43.23, 296.2, 296.20, 296.21, 296.22, 296.23,296.24, 296.25, 296.26, 296.3, 296.30, 296.31, 296.32, 296.33, 296.34, 296.35, 296.36, 296.82, 298.0, 300.4, 301.12, 309.0, 309.00, 309.1, 309.28, 311, 311.0, 311.00 |
| Low birth weight (<5 lbs 8 oz) and less than 24 months old | P07.10, P07.00 |
| Oral health problems | K00* through K14* |
| Recent Major Surgery, trauma, burns | S00* through T88* |
| Infectious Diseases | A00* through B99 |
| Gastrointestinal Disorders | K00* through K95* |
| Genetic/Congenital Disorders | Q00* through Q99 |
| Low iron | E61.1 |

**Supplemental Table 3.** Early Intervention Eligibility Proxy

| Age Group Eligibility Criteria | ICD-10 Diagnosis Codes |
| --- | --- |
| Newborn – birth to 30 days |  |
| Very low birth weight (i.e., less than 1,500 grams) | P07.15,  P07.14 |
| Chronic lung disease (bronchopulmonary dysplasia) | P27.1 |
| Neonates, Infants, Toddlers |  |
| Chromosomal conditions | Q90-Q99 |
| Blindness, including visual impairments | H54 |
| Down Syndrome | Q90.0 |
| Deafness, including hearing impairments | H91.90 |
| Epilepsy/seizure disorder | G40 |
| Autism spectrum disorder | F84.0 |
| Blood lead level of five micrograms per deciliter or greater | R78.71 |
| Cranio-facial anomalies |  |
| Cyanotic congenital heart disease | [I24.9](https://www.icd10data.com/ICD10CM/Codes/I00-I99/I20-I25/I24-/I24.9), Q24.9 |
| Neonatal abstinence syndrome | P96.1 |

**Supplemental Table 4.** LASSO Model Coefficients

| Predictor | With Race | Without Race |
| --- | --- | --- |
| Functional Neonatal Abstinence Syndrome | 1.765 | 1.725 |
| OOHP AFK flag (past 3 months) | 0.678 | 0.621 |
| OOHP Eligibility flag (past 12 months) | 0.585 | 0.595 |
| Functional Substance Use Disorder | 0.549 | 0.480 |
| OCOI: Family stability, quintile 3 | 0.481 | 0.469 |
| OOHP Eligibility flag (past 3 months) | 0.389 | 0.374 |
| OCOI: Family stability, quintile 2 | 0.315 | 0.307 |
| OCOI: Access, quintile 3 | 0.305 | 0.305 |
| OCOI: Environment, quintile 2 | 0.293 | 0.228 |
| OCOI: Housing, quintile 1 | 0.252 | 0.248 |
| OCOI: Access, quintile 1 | 0.234 | 0.098 |
| PMCA: Renal claims | 0.217 | 0.210 |
| Comprehensive behavioral health definition | 0.127 | 0.106 |
| OCOI: Education, quintile 3 | 0.099 | 0.149 |
| PMCA: Progressive claims | 0.094 | 0.059 |
| Race: Black or African American | 0.036 |  |
| Inpatient LOS (past month) | 0.021 | 0.019 |
| Inpatient LOS (past 3 months) | 0.014 | 0.014 |
| OCOI: Infant health, quintile 4 | 0.010 |  |
| USDA: Low food access, 1 and 20 | 0.009 |  |
| Gender: Male | 0.004 |  |
| Inpatient LOS (past 6 months) | 0.003 | 0.002 |
| OCOI: Family stability, quintile 5 | -0.015 | -0.043 |
| PMCA: Hematology claims | -0.030 |  |
| PMCA: Immunology claims | -0.035 |  |
| PMCA: Malignancy claims | -0.036 |  |
| OCOI: Family stability, quintile 1 | -0.039 |  |
| PMCA: Mental health claims | -0.053 | -0.031 |
| USDA: HUNV | -0.081 |  |
| Age (years) | -0.091 | -0.087 |
| OCOI: Children's health, quintile 4 | -0.096 | -0.103 |
| PMCA: Cardiac claims | -0.105 | -0.036 |
| PMCA: Pulmonology/respiratory claims | -0.107 | -0.083 |
| PMCA: Genito. claims | -0.117 | -0.088 |
| OCOI: Education, quintile 2 | -0.119 |  |
| Ohio Early Intervention | -0.175 | -0.140 |
| PMCA: Musculo. Claims | -0.207 | -0.163 |
| PMCA: Opthal. Claims | -0.248 | -0.218 |
| OCOI: Housing, quintile 3 | -0.263 | -0.137 |
| OCOI: Infant health, quintile 2 | -0.282 | -0.291 |
| OOHP AFK flag (past 6 months) | -0.295 | -0.289 |
| PMCA: Endocrinology claims | -0.298 | -0.219 |
| OCOI: Housing, quintile 4 | -0.309 | -0.343 |
| OOHP elig. flag (past 6 months) | -0.323 | -0.306 |
| USDA: Low food access, 1/2 and 10 | -0.360 | -0.356 |
| OCOI: Infant health, quintile 5 | -0.401 | -0.453 |
| OCOI: Environment, quintile 3 | -0.685 | -0.710 |
| Race: Native American | -0.822 |  |
| OCOI: Education, quintile 5 | -0.840 | -0.714 |
| Race: Other/Unknown | -1.005 |  |
| OCOI: Access, quintile 5 | -1.133 | -1.049 |
| OCOI: Access, quintile 4 | -1.992 | -1.818 |
| Race: Asian | -2.106 |  |
| Model Intercept | -5.260 | -5.438 |

OOHP: Out-of-home placement

OCOI: Ohio Children’s Opportunity Index

PMCA: Pediatric Medical Complexity Algorithm

LOS: Length of stay

HUNV: Percent of housing units with no vehicle


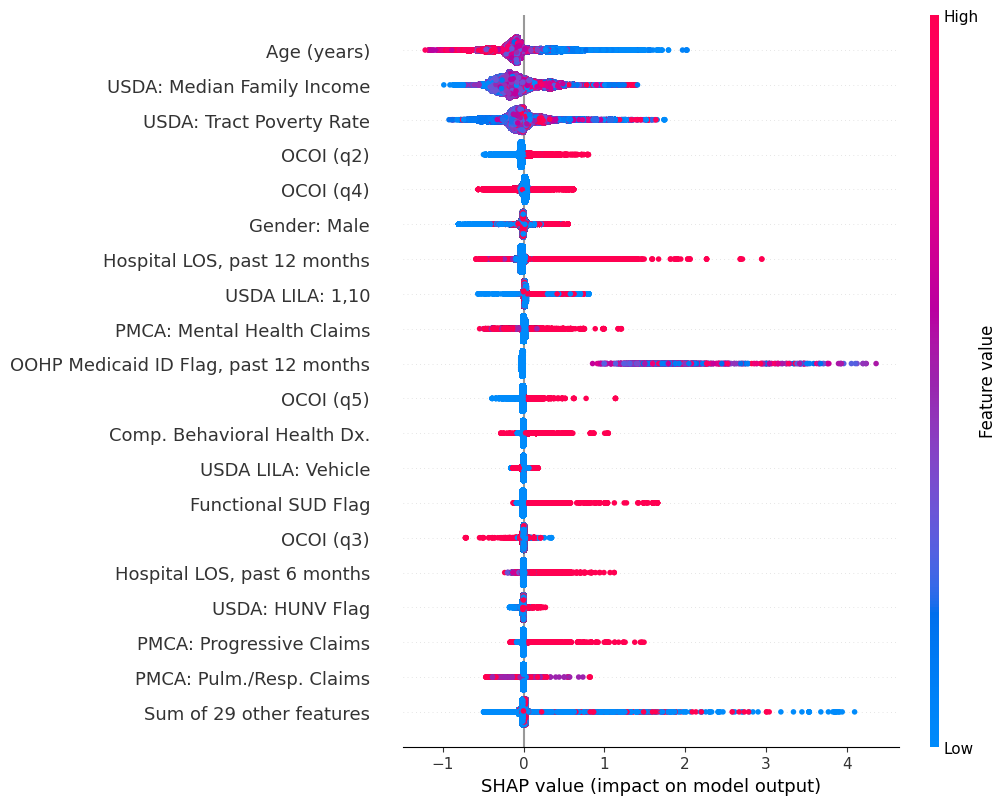


**Supplemental Figure 1.** XGBoost Shapley additive explanation (SHAP) values without race as a candidate predictor. Higher predictor values are shaded red, with lower values shaded blue. For binary predictors, such as “Gender: Male” and OCOI quintiles, positive observations are shaded red. An example interpretation is that younger age (blue shades of “Age (years)”) generally contributes positively to the predicted probability of out-of-home placement while the positive (red) Functional SUD Flag indicates increased predicted probability of out-of-home placement.

OCOI: Ohio Children’s Opportunity Index quintiles

LOS: Length of stay

LILA: Low-income and low-access (food) tracts

PMCA: Pediatric Medical Complexity Algorithm

OOHP: Out-of-home placement

SUD: Substance use disorder

HUNV: Percent of housing units with no vehicle


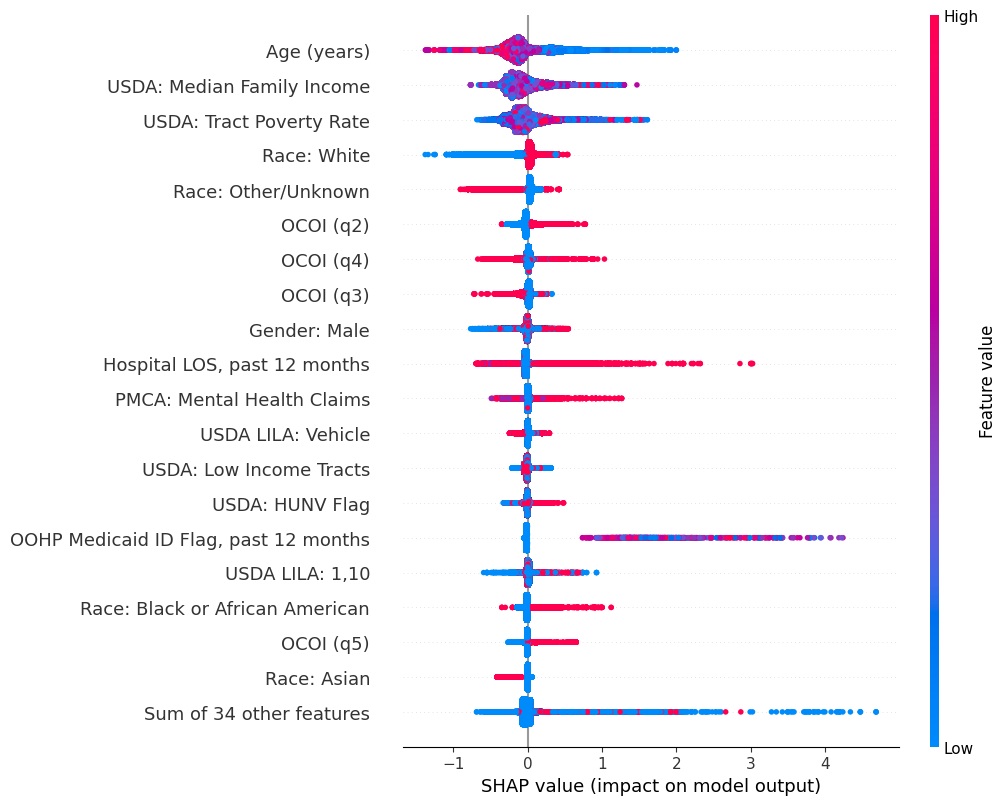


**Supplemental Figure 2.** XGBoost Shapley additive explanation (SHAP) values with race as a candidate predictor. Higher predictor values are shaded red, with lower values shaded blue. For binary predictors, such as “Race: White” and OCOI quintiles, positive observations are shaded red. An example interpretation is that younger age (blue shades of “Age (years)”) generally contributes positively to the predicted probability of out-of-home placement while the positive (red) Functional SUD Flag indicates increased predicted probability of out-of-home placement.

OCOI: Ohio Children’s Opportunity Index

PMCA: Pediatric Medical Complexity Algorithm

LILA: Low-income and low-access (food) tracts

HUNV: Housing Units with no vehicle

OOHP: Out-of-home placement
